# Comparison of two different suture techniques in laparoscopic partial nephrectomy

**DOI:** 10.1590/S1677-5538.IBJU.2016.0550

**Published:** 2017

**Authors:** Onur Kaygisiz, Sinan Çelen, Berna Aytac Vuruşkan, Hakan Vuruşkan

**Affiliations:** 1 Department of Urology, Uludag University, Faculty of Medicine, Bursa, Turkey; 2 Afyon Sandikli Government Hospital, Afyon, Turkey; 3 Department of Surgical Pathology, Faculty of Medicine, Uludag University, Bursa, Turkey

**Keywords:** Nephrectomy, Laparoscopy, Suture Techniques

## Abstract

**Objective:**

To comparatively evaluate the traditional interrupted knot-tying and running suture renorrhaphy with Monocryl^®^ in laparoscopic partial nephrectomy (LPN).

**Materials and Methods:**

A retrospective analysis of 62 consecutive patients undergoing LPN using traditional interrupted knot-tying suture renorrhaphy (Group 1; n=31) or running suture technique renorrhaphy with 2-0 monofilament polyglecaprone (Monocryl^®^, Ethicon) (Group 2; n=31) from December 2011 to October 2015 at the University. All patients underwent LPN performed by an experienced laparoscopic surgeon. The demographic, perioperative and postoperative parameters were compared between the groups, and the effect of both suture techniques on the warm ischemic time (WIT) and trifecta were evaluated.

**Results:**

The running suture renorrhaphy with Monocryl^®^ reduced WIT, estimated blood lost and length of hospitalization stay significantly without increasing postoperative complication rate during LPN in comparison with interrupted knot-tying suture.

**Conclusion:**

The renorrhaphy using the running suture with Monocryl^®^ is an effective and safe technique with the advantage of shortening WIT even in more challenging and larger tumors during LPN.

## INTRODUCTION

Partial nephrectomy (PN) is recommended for localized renal cancers as an alternative to radical nephrectomy (1). Previous studies reported less renal function loss and decreased risk of chronic kidney disease after PN than radical nephrectomy (2, 3). The laparoscopic approach in PN has become more common in recent years due to the advantages of lower morbidity, shorter hospitalization stay, reduced postoperative pain, and increased patient satisfaction compared to the open approach (4).

The success of the PN technique is based on the negative surgical margin for oncological outcome and the minimum warm ischemia time (WIT) for renal function preservation (5). Laparoscopic PN (LPN) and open PN have similar outcomes in terms of positive surgical margin, but LPN has longer WIT compared to open PN (6, 7) due to technical and ergonomic challenges of laparoscopic suturing. A recent study reported that increased WIT plays an important role in renal functional loss in the early postoperative period in elective LPN (8).

It is important to determine the laparoscopic suture technique in PN that minimizes WIT. Different suture materials and techniques have been suggested to speed up the reconstruction under warm ischemia (9-12). A limited number of studies compared the suture techniques using PDS II, self-retaining barbed sutures, and polyglactin sutures (8, 13).

However, to our knowledge, no study has compared suture techniques using poliglecaprone (Monocryl^®^; Ethicon) for running suture renorrhaphy. Thus, the aim of this study was to comparatively evaluate two alternative renorrhaphy techniques in laparoscopic PN using Monocryl^®^.

## MATERIALS AND METHODS

Medical data from a series of 143 consecutive patients treated with LPN were evaluated between December 2011 and October 2015. The selected patients were operated on by a single experienced laparoscopic surgeon (HV). Sixty-six patients who were operated on by different surgeons were excluded. Ten patients who were followed up for less than 6 months were also excluded, as well as five patients with a solitary kidney, other cancers, or history of kidney operation. The remaining 62 consecutive patients were included in the study. LPN was performed by traditional interrupted knot-tying suture renorrhaphy (Group 1, n=31), and the last 31 patients were treated with the running suture technique using Monocryl^®^ (Group 2).

All patients were assessed with whole blood count, blood chemistry, and triphasic computed tomography. Preoperative variables included age, gender, body mass index (BMI), Charlson Comorbidity Index, size and location of renal mass, hematocrit (HCT) value, and creatinine value. Perioperative and postoperative parameters included the WIT, operative time, estimated blood loss (EBL), transfusion rate, complications, and length of hospital stay (LOS). Intraoperative and postoperative complications were classified according to SATAVA and Clavien-Dindo classifications (14, 15).

Both groups were followed up for a median of 7 months for the evaluation of renal function and cancer status after LPN. Renal function was assessed by eGFR using the Modification of Diet in Renal Disease (MDRD) study equation (16)). The R.E.N.A.L. Nephrometry Score was analyzed by preoperative abdominal triphasic computed tomography by one specialist (17). Trifecta was defined as a combination of negative surgical margin, WIT less than 25 minutes, and zero perioperative complications (18). To test the reproducibility of the assessments of scores, the same examiner reassessed all computed tomography scans two weeks after the first evaluation. The differences between double interpretations were statistically tested.

### Surgical technique

Transperitoneoscopic PN was performed in all patients. All surgeries were performed by the same experienced laparoscopic surgeon. All patients were placed in a lateral decubitus position. The pneumoperitoneum was established using the Veress technique. After insufflation of the abdomen, three or four ports were placed. After the renal mass was localized, Gerota's fascia was dissected, leaving the tumor with overlying perinephric fat. The renal artery was dissected and mobilized gently. The renal capsule around the mass was incised to mark the position. After mannitol administration, the renal artery was cross-clamped using a Satinsky clamp or bulldog clamp. The renal vein was not clamped. The tumor was excised with cold scissors from the kidney. The renal mass was placed in an endobag at the end of the surgery.

In Group 1, the interrupted suture technique was performed with 2-0 polypropylene suture (Prolene^®^; Ethicon, Somerville, NJ) and 3-0 polyglactin 910 suture (Vicryl^®^; Ethicon, Somerville, NJ). The blood vessels were repaired with 2-0 Prolene^®^, and nodes were done separately for each vessel. If there was an opened pelvicalyceal system, it was sutured with using 2-0 Vicryl^®^ by interrupted suture closure with knot tying. Then, Surgicel^®^ (Ethicon Endo-Surgery, Somerville, NJ) was placed on the parenchyma defect site. The renal parenchymal defect suture was performed using 2-0 Vicryl^®^ over the Surgicel^®^ by interrupted suture closure with knot tying. After the suture closure was completed, the bulldog or Satinsky clamp was removed.

In Group 2, 2-0 Monocryl^®^ (poliglecaprone 25; Ethicon) suture was used. A Hem-o-lok^®^ clip was applied to the suture's terminal end by cutting to a length of 20cm. If there was an opened pelvicalyceal system, it was firstly closed with 3-0 polyglactin 910 (Vicryl; Ethicon). After the initial suture using prepared 2-0 Monocryl^®^ suture (poliglecaprone 25; Ethicon), the needle was passed from outside to inside through the renal parenchyma. The tumor bed was then sutured two or three times using running sutures.

At the end, the suture was removed from the renal capsule, tension was applied, and the suture was locked with Hem-o-lok^®^ clips (Teleflex^®^ Medical, Research Triangle Park, NC). The clamps were removed, and the tumor bed was inspected to ensure hemostasis. If necessary, additional sutures were supplied or only one Surgicel^®^ (Ethicon Endo-Surgery, Somerville, NJ) was placed in the tumor bed. The outer parenchymal layer was repaired as described above after the clamps were removed.

### Statistical analysis

The data were statistically analyzed by using SPSS 23.0 (SPSS Inc, Chicago, Ill, USA). The variables were compared according to groups. The Shapiro-Wilk test was applied to test the normality of continuous variables. The normally distributed variables are presented as the mean±standard deviation and compared using a student's t-test. The non-normally distributed variables are presented as the median (minimum-maximum) and compared using the Mann-Whitney U test. The Wilcoxon signed rank test was used for non-normally distributed related samples. Nominal data are presented as a number or percentage and compared using the chi-square test. A p-value less than 0.05 was considered as statistically significant.

## RESULTS

The mean age was 57.8±19.5 years. There were no significant differences between the groups with respect to age, gender, BMI, Charlson Comorbidity Index, preoperative HCT level, preoperative creatinine, preoperative eGFR, or chronic kidney failure rate (Table-1). The intraexaminer correlation coefficient for repeated scores was 0.93, indicating high reliability. The locations and pathology of the renal mass and R.E.N.A.L. Nephrometry Score were found to be similar in both groups, although the renal mass size was significantly higher in Group 2 (Table-1). Renal mass sizes were 3.0±1.03 cm and 3.97±1.47cm for Groups 1 and 2, respectively (Table-1). The median R.E.N.A.L. Nephrometry Scores were 4 (4-6) and 5 (4-10) for Groups 1 and 2, respectively (Table-1). The positive surgical margin rate did not differ between the groups. Positive surgical margins were 2 and 1 for groups 1 and 2, respectively (p=0.612). None of the patients showed progression during a median of 36 months of follow-up.

**Table 1:** Comparison of demographic, perioperative, and pathological outcomes between interrupted knot-tying suture renorrhaphy and running suture renorrhaphy with polyglecaprone

Age		Group 1	Group 2	p
		53 (32-78)	59 (38-80)	0.123
Gender (Female/Male)		9/22	13/18	0.426
Charlson Comorbidity Index		3 (2-5)	3 (2-5)	0.052
BMI		27.21 (17-47)	27.12 (22-47)	0.833
Preoperative Hct		14.2 (10.3-18)	13.0 (10.3-16.8)	0.058
Preoperative creatinine		0.8 (0.6-2.8)	0.7 (0.6-1.9)	0.250
Preoperative eGFR (mL/min/1.73m^2^)		93.77±24.74	92.81±27.60	0.885
**CKD 3A or more**	3A	1	0	
	3B	1	2	0.597
	4	1	1	
**Side (right)**		54.5%	45.5%	0.611
**Localization**	Upper	9	8	
	Middle	11	11	0.950
	Lower	11	12	
**Pathology of renal mass**	Clear Cell RCCa	10	14	
	Chromophobe RCCa	6	1	0.804
	Papillary RCCa	7	8	
	Multicystic RCCa	2	2	
	Benign	6	6	
**Mass size (cm)**		3 (2-5)	4 (2-8)	0.006
**RENAL score**		4 (4-6)	5 (4-10)	0.068

**CKD** = Chronic Kidney Disease stage

The median WITs were 21 (13-42) and 13 (6-26) minutes for Groups 1 and 2, respectively, and it was significantly lower in Group 2 than in Group 1 (p<0.001). WIT was over 25 minutes in six cases in Group 2 and in only one case in Group 1. The HCT differences and red blood cell transfusion rates were similar in both groups on the first day, but EBL was higher in Group 1 (Table-2). The perioperative opened pelvicalyceal system rate and operative time were similar between groups (Table-2). There were no significant differences between the groups with regard to perioperative and postoperative complications (Table-2). In Group 1, diaphragmatic rupture was treated with laparoscopy in the same season by the same surgeon. In Group 2, one patient underwent angiography one week after the operation for late hemorrhage. None of the cases were converted to open surgery. However, LOS was lower in Group 2 (2.55±0.96 days) than in Group 1 (3.23±1.26 days; p=0.02, Table-2). Trifecta rates were similar in both groups.

**Table 2:** Comparison of perioperative and postoperative outcomes between groups.

		Group 1	Group 2	p
WIT (min)		21 (13-42)	13 (6-26)	<0.001
WIT ≥25 min		6 (19.4%)	1 (3.2%)	0.052
Positive surgical margin		2 (6.5%)	1 (3.2%)	0.612
Operative time (min)		120 (60/210)	120 (60/210)	0.656
Estimated blood loss		85 (30-300)	50 (30-450)	0.010
Δ Hct 1		-1.6 (-5.2/0.10)	-1.7 (-4.6/0.2)	0.330
Δ Creatinine day 2		-0.1 (-0.7/0.1)	0 (-0.6/0)	0.877
Δ Creatinine month 6		0 (-0.9/0.3)	0 (-0.2/0.1)	0.719
eGFR month 6 (mL/min/1.73m^2^)		104 (18-125)	95 (26-133)	0.481
ΔeGFR6 (mL/min/1.73m^2^)		-7 (-51/19)	-6 (-31/17)	0.619
ΔeGFR6 (mL/min/1.73m^2^)		0(-20/39)	0 (-4/8)	0.680
**SATAVA**	1	5 (16.1%)	4 (12.9%)	
	2A	3 (9.7%)	3 (9.7%)	0.744
	3	0	1 (3.2%)	
**Trifecta rate**		71%	80.6%	0.554
**Complication Clavien**	1	5 (16.1%)	6 (19.4%)	0.809
	2	2 (6.5%)	1 (3.2%)	
LOS (day)		3 (2-7)	2 (2-6)	0.008

Δ **Creatinine 6** - changes in creatinine in postoperative 6 month from baseline

Δ **Hct 1** - changes in hematocrit creatinine in postoperative first day

The median serum creatinine levels were significantly increased and eGFR was significantly decreased after LPN in both Groups. However, there were no significant differences between the preoperative values and 6-month follow-up in both Groups (Table-3). The creatinine and eGFR differences were similar in both Groups (Table-2).

**Table 3:** Renal function changes overtime between groups.

		Preoperative	Postoperative day 2	Postoperative month 6	p1	p2
Median Creatinine level	G1	0.8 (0.6-2.8)	0.8 (0.6-3.5)	0.8 (0.6-3.7)	0.02	0.929
	G2	0.7 (0.6-1.9)	0.8 (0.6-2.4)	0.7 (0.6-2.0)	0.01	0.414
eGFR (mL/min/1.73m^2^)	G1	100 (25-138)	93 (18-117)	104 (18-125)	0.014	0.221
	G2	95 (29-133)	90 (22-115)	95 (26-133)	0.03	1

**p1** = statistical significance between preoperative and postoperative 2 day value; **p2 =** statistical significance between preoperative and postoperative 6 month value. **G1 =** Group 1; **G2 =** Group 2

## DISCUSSION

LPN is a minimally invasive approach for localized renal tumors (1). It has shown comparable oncologic outcomes to open PN while providing shorter hospital stay, decreased convalescence, and reduced pain (4). Despite the benefits of LPN, it is a technically demanding procedure with a higher rate of intraoperative complications and longer WIT compared to open surgery (19). Becker et al. (20) emphasized that WIT is the strongest modifiable surgical risk factor for postoperative chronic kidney disease. Lane et al. (19) also reported that the major surgical factor in renal function was WIT among the patient-specific, tumor-specific, and surgical factors after PN. Funahashi et al. (21) found that a WIT of 25 minutes or more caused irreversible damage distributed diffusely throughout the operated kidney. Thompson et al. (22) reported that with prolonged WIT, ischemia reperfusion was positively associated with short- and longterm renal consequences, and they suggested that every minute counts for the severity of damage when the renal hilum is clamped. Therefore, WIT should be shortened as much as possible to help preserve renal function (20, 22).

Link et al. (23) stated that longer ischemia time in LPN is likely to result in difficulty in renorrhaphy with laparoscopy. Intracorporeal suturing was the most time-consuming stage during LPN. Thus, simplifying the suture technique could reduce WIT and better preserve renal function. There are limited numbers of studies considering the effects of simplifying the suture technique on WIT, and none of them used polyglecaprone sutures (9-13). Therefore, the present study aimed to investigate the efficacy of renorrhaphy using running suture technique with monofilament polyglecaprone (Monocryl^®^, Ethicon) on reducing renorrhaphy time and WIT during LPN in comparison with interrupted knot-tying suture renorrhaphy.

The main goals of PN are providing WIT less than 25 minutes, as suggested by Thompson et al. (22), as well as negative surgical margins for oncological safety without complications defined as trifecta (18). In this study, the trifecta rate was similar between groups, but WIT was significantly reduced in the group undergoing running suture technique renorrhaphy with Monocryl^®^. Erdem et al. (12) reported a significantly reduced WIT of 9 minutes in a group treated by self-retaining barbed suture rather than polyglactin suture. Jeon et al. also reported a shorter WIT of 7.4 minutes in a barbed suture group than a polyglactin suture group (24). The advantage of self-retaining barbed suture (V-Loc) in decreasing WIT might result from passing through the tissue in only one direction, preventing the suture from slipping and eliminating the need to maintain continuous tension while suturing and tying knots. In another study, the mean WIT was 21.5 minutes in running suture renorrhaphy using PDS suture, while it was 32.3 minutes in the interrupted suture group (13).

PDS II^®^ suture was reported to be 1.4 times as stiff as Monocryl^®^ suture (25). Bezwada et al. (25) showed that the lower stiffness and pliability of Monocryl^®^ suture resulted in excellent handling and tensile properties with minimal resistance during passage through tissue, and Monocryl^®^ had very good tactile feedback. Vicryl^®^ is a braided multifilament suture that causes resistance when passing through the kidney during LPN. Monocryl^®^ suture stretches more than Vicryl^®^ and PDS at higher loads (26). In this study, Monocryl^®^ suture was preferred for providing faster parenchymal suturing with minimal tissue damage, and it is also more cost-effective than V-Loc.

The median WIT was reduced 8 minutes in patients treated with running suture using Monocryl^®^ than traditional renorrhaphy, despite larger tumors in running suture renorrhaphy Group. Likewise, WIT was higher than 25 minutes for only one patient in Group 2 but for 6 patients in Group 1. During running suture renorrhaphy, the surgeon does not need to see the bleeding vessels clearly to control the hemorrhage after unclamping. Thus, this technique facilitates renal parenchymal suturing and gives confidence to the surgeon for unclamping before repairing the renal parenchymal defect. These features also contribute to decreasing WIT.

The postoperative decline of renal function after LPN was recovered to preoperative baseline values after 6 months post-operation in both groups. There were no significant differences between the groups with regard to renal function. WIT was significantly lower in Group 2. The median WIT was 21 minutes in Group 1, and only 6 patients had WIT ≥25 minutes, which could be a reason for the similarity of renal function.

HCT difference from the first day, transfusion rate, operative time, perioperative and postoperative complications, and trifecta rate were similar in both Groups. However, EBL was found to be higher in Group 1 than in Group 2. Olweny et al. (27) reported that barbed sutures reduce the incidence of serious intraoperative bleeding. Similar results were observed with other V-Loc series (24, 28). Our results are in accordance with these studies. In Group 2, one patient underwent angiography one week after operation for late hemorrhage.

After minimally invasive PN, Omea et al. (29) found an unexpectedly high rate of 21.7% for asymptomatic unruptured renal artery pseudoaneurysm detected by computed tomography arteriography in the early period. However, in a systematic review and comparative analysis, Jain et al. (30) reported that the rate of symptomatic pseudoaneurysm was 1.96%. In our study, only one patient in Group 2 underwent angiography one week after the operation for symptomatic pseudoaneurysm. This ratio represents 1.6% of the total patients, which is in accordance with the literature. It is hard to make a conclusive decision about the comparison of the groups with regard to pseudoaneurysm due to the limited number of the patients.

LOS was shorter in the running suture renorrhaphy Group than the traditional renorrhaphy Group. Running suture renorrhaphy provides a decline of 0.7 days in mean LOS. However, this study cannot provide a definite conclusion about this issue.

One of the limitations of this study is its retrospective nature. However, both Groups were treated by the same surgeon, who had experience with at least 1.000 laparoscopic surgeries, including radical nephrectomy, PN, radical prostatectomy, and radical cystectomy. The same experienced surgeon used the standard surgical technique. Secondly, renal mass, was smaller in the traditional renorrhaphy Group. However, WIT was longer in the traditional renorrhaphy group, although renal mass was smaller. Thirdly, the sample size was small, and the results should be confirmed with more cases.

## CONCLUSIONS

Renorrhaphy using running sutures with Monocryl^®^ is an effective and safe technique that shortens WIT. EBL and LOS are also decreased with this technique.
